# Effect of Supervised Students' Involvement on Diagnostic Accuracy in Hospitalized Medical Patients — A Prospective Controlled Study

**DOI:** 10.1371/journal.pone.0044866

**Published:** 2012-09-11

**Authors:** Dorothea Adelheid Herter, Robert Wagner, Friederike Holderried, Yelena Fenik, Reimer Riessen, Peter Weyrich, Nora Celebi

**Affiliations:** 1 Medical School, University of Tuebingen, Tuebingen, Germany; 2 Department for Endocrinology, Diabetology, Nephrology and Angiology, University Hospital of Tuebingen, Tuebingen, Germany; 3 Office of the Dean, Medical School, University of Tuebingen, Tuebingen, Germany; 4 Medical Intensive Care Unit, Tuebingen, Germany; University of Washington, United States of America

## Abstract

**Background:**

During internships most medical students engage in history taking and physical examination during evaluation of hospitalized patients. However, the students' ability for pattern recognition is not as developed as in medical experts and complete history taking is often not repeated by an expert, so important clues may be missed. On the other hand, students' history taking is usually more extensive than experts' history taking and medical students discuss their findings with a Supervisor. Thus the effect of student involvement on diagnostic accuracy is unclear. We therefore compared the diagnostic accuracy for patients in the medical emergency department with and without student involvement in the evaluation process.

**Methodology/Principal Findings:**

Patients in the medical emergency department were assigned to evaluation by either a supervised medical student or an emergency department physician. We only included patients who were admitted to our hospital and subsequently cared for by another medical team on the ward. We compared the working diagnosis from the emergency department with the discharge diagnosis. A total of 310 patients included in the study were cared for by 41 medical students and 21 emergency department physicians. The working diagnosis was changed in 22% of the patients evaluated by physicians evaluation and in 10% of the patients evaluated by supervised medical students (p = .006). There was no difference in the expenditures for diagnostic procedures, length of stay in the emergency department or patient comorbidity complexity level.

**Conclusion/Significance:**

Involvement of closely supervised medical students in the evaluation process of hospitalized medical patients leads to an improved diagnostic accuracy compared to evaluation by an emergency department physician alone.

## Introduction

History taking is one of the most valuable tools in making a diagnosis during the evaluation of a patient [1]. This has not changed over time and with growing experience physicians tend to appreciate the information gathered during history taking even more [2]. Some investigations focused on the quantitative contribution of history taking in making the final diagnosis. In a study by Hampton on 80 outpatients, the final diagnosis was correct after reading the referral letter and taking a history in 82% of the cases [3]. Leuppi et al. investigated the contribution of history taking in 243 patients with chest symptoms and found that the diagnosis was correct after history taking in 41% of the cases while physical examination did not improve the diagnostic accuracy [4]. Alboni et al. investigated the diagnostic value of history taking in 341 patients with syncope and found that the history of heart disease predicted a cardiac cause of syncope with a sensitivity of 95% and a specifity of 45%, while the absence of cardiac disease excluded a cardiac cause with 97% accuracy [5]. However, not only experienced physicians take histories; this task is often delegated to medical novices or young doctors at the beginning of their career.

Medical novices and experts fundamentally differ in interpreting information gathered during history taking [6], [7]. Medical experts develop an ability to identify meaningful patterns and features (schemas) in the given information resulting in non-analytical reasoning [8], [9], [10], [11], [12]. In addition, experts have a more extensive and better organized knowledge than novices and a better selection between relevant and irrelevant information, while students usually have better memories [13], [14], [15]. Experts can usually flexibly switch between the analytical and the non-analytical reasoning mode in making the diagnosis [16]. Novices employ non-analytical reasoning with inferior accuracy and have to rely on analytical reasoning as the primary tool for making the diagnosis [16], [17]. There is a vast body of evidence, that, given the same information and in controlled laboratory settings, the medical expert outperforms the novice in making the correct diagnosis based on history alone [11], [15], [16]. In addition, asking further questions is warranted during history taking, since the information that patients volunteer is often incomplete. In a study by Scheitel et al., patients failed to report 68% of their health problems [18]. It is unclear, whether novices are able to identify information gaps in the statements volunteered by the patients.

Most medical schools worldwide require clerkships and internships for their medical students [19], [20], [21], [22], [23], [24], [25]. During this practical training medical students usually engage in history taking and physical examination. This sometimes cumbersome task is often not completely repeated by an experienced physician. So, when medical students take a patient's history, important clues may be missed.

However, in the clinical setting, more factors need to be considered. Student's history taking is generally much more extensive than history taking by a physician. In addition, medical students can usually take a history without much distraction while medical experts have to handle several tasks at once and are under greater time pressure [26], [27]. Laxmisan et al. reported an interruption every 9–14 minutes for physicians in the emergency department [28]. Multitasking and interruptions may impair the quality of the history taking [29]. In a study by Westbrock et al., frequent interruptions lead to a shortening of the time spent on a single task and a substantial risk that the task was abandoned altogether [30].

In our experience, medical students usually perform history taking and physical examination with fewer interruptions compared to physicians. In addition, students usually do not work alone; they should be supervised to some degree and the diagnosis is based on the results of history taking, physical examination and additional information gathered from laboratory data and imaging studies [31], [32].

The effect of student involvement in the evaluation process on the diagnostic accuracy in the clinical setting is unclear. Since history taking is probably most important for sick and multimorbid medical patients, we investigated the effect of students' involvement in the evaluation of medical patients, who were admitted from the emergency department to the clinical decision unit or to the ward and thus were treated for the same problem by two different medical teams. We conducted a prospective, controlled study in order to compare diagnostic accuracy defined as the congruence between working diagnosis from the emergency department with the final diagnosis from the hospital's discharge letter, with medical student involvement in the evaluation process in the medical emergency department.

## Results

A total of 323 patients who presented to the medical emergency department during the study were admitted for further treatment, 13 patients had to be excluded due to insufficient documentation, ten of these patients were seen by the emergency department physician and three by the supervised medical students. The emergency department physician saw 172 of the remaining 310 patients and the medical students who were supervised by another emergency department physician (Supervisor) saw 138 patients.

The characteristics of the patients are listed in table 1 according to the two different groups (seen by the supervised medical student or the emergency department physician).

**Table 1:** Characteristics of the patients seen by supervised medical students and emergency department physicians.

	All	Supervised medical student	emergency department physician	p
n	310	138 (44.5%)	172 (55.5%)	
Diagnostic accuracy	83%	90%	78%	.003
Age	65.4 (±15.8) years	67.8 (±15.7) years	63.5 (±15.7) years	.005
Gender	male 165 (53.3%), female 145 (46.7%)	male 74 (53.6%), female 64 (46.4%)	male 91 (52.9%), female 81 (47.1%)	.9
PCCL 0	0: 115	0: 45	0: 70	.5
PCCL 2	51	24	27	.5
PCCL 3	68	31	37	.5
PCCL 4	76	38	38	.5
Length of stay in the ED	329 (±150) min	317 (±131) min	339 (±163) min	.4
Expenditures on diagnostic procedures	36.1 (±56.9) €	37.4 (±59.7) €	35.1 (±54.8) €	.6

P values were calculated using the Wilcoxon-test for numeric data and the Fisher's exact test for non-numeric data. Diagnostic accuracy: congruence between working diagnosis from the Emergency department and final diagnosis from the discharge letter. PCCL: patient comorbidity complexity level from the national Diagnosis related Group system. Expenditures on diagnostic procedures: calculated with the official national medical fee schedule for hospitals.

The physician team on the ward changed the working diagnosis from the emergency department in 22% (38/172) of the patients with history taking by an emergency department physician and 10% (14/138) of the patients with history taking by a supervised medical student (p.006). The supervised medical students saw older patients (67.8±15.7 years versus 63.5±15.7 years, p = .005).

There was no significant difference in length of stay, expenditures for diagnostic procedures or Patient-Comorbidity-Complexity-Level, which constitutes an established measure of disease complexity with corresponding diagnostic expenditure (see table 1; all p>.05).

## Discussion

Medical students in clerkships engage in history taking, one of the most valuable tools for making the diagnosis that is often not repeated by an experienced physician. Therefore we investigated the effect of student involvement in the evaluation process of hospitalized medical patients on the diagnostic accuracy.

In our study the diagnostic accuracy was better, when supervised medical students were involved while there was no difference in the main patient characteristics (apart from age – the students saw older patients) and in resources used to find the diagnosis. Several factors may have contributed to this finding.

Firstly, the working conditions for the medical students in our setting were far better than for the physicians. An emergency department physician saw approximately three times as many patients per day as the average medical student in our study. To make sure that medical students meet their learning goals at the end of their internship, our faculty members were encouraged to give students enough time to assess their patients without interruptions. Therefore medical students in our setting could fully concentrate on the patient without many distractionsor interruptions, while the physician working alone had to “run” the emergency department: handle several patients at once, deal with the telephone calls, answer the questions of the staff, do the organization, and so on. In controlled laboratory conditions with equal working conditions and equal time for history taking for both groups the results would probably have been different [16]. Interestingly, the diagnostic accuracy of the physicians in our study (78%) corresponded approximately with the diagnostic accuracy Dormann et al. reported for their emergency department (71%), while the students' diagnostic accuracy (90%) corresponded with diagnostic accuracy Heuer et al. reported for emergency physicians in the pre-hospital setting (90%) [33], [34]. In the pre-hospital setting, one physician usually sees only one patient at a time. Therefore, one might assume that the students did not outperform the physicians in interpreting the gathered data, but that the physicians could not tap their full potential in data acquisition owing to the working conditions present in the typical emergency department setting. The degree of completeness of data predict the diagnostic accuracy regardless of clinical experience [35]. Indeed, the nature of the diagnosis mismatched by the emergency department physician (e.g. chest pain was attributed to acute coronary syndrome, which was not confirmed later on, dyspnea was attributed to atrial fibrillation while later diagnostics revealed pneumonia) might suggest premature closure of the diagnostic reasoning process – although our study was not designed to investigate the cognitive reasoning processes.

Secondly, in our setting, the diagnosis in the student group was the result of a group discussion between the student mainly responsible for the patient's evaluation process, the other students present on that day and their Supervisor, so there was combined mental power including the non-analytical reasoning of the Supervisor. The working diagnosis formulated by the emergency department physician working alone was not challenged in a similar manner. The superiority of teams compared to individuals in decision making is a well known phenomenon which has been demonstrated in several clinical contexts [36], [37]. Coderre et al. showed that questioning a diagnosis by providing additional information helps medical students to correct their initially incorrect diagnosis and leads to a higher diagnostic accuracy [38]. This might support our assumption that discussing the patient's case with the Supervisor was one of the reasons for higher diagnostic accuracy in this team. Furthermore, there is evidence that two separate assessments may prevent extreme rating [37]. A medical team (regardless of whether students or physicians) can certainly benefit from combining the team members' knowledge.

However, our study has several limitations. First of all, our findings only reflect single center experiences in one emergency department in Germany and only for sick and rather multimorbid medical patients. It would be interesting to examine the effect of students' involvement in different contexts, different health care systems and in different hospitals.

Secondly, we could not blind all the physicians involved. All medical students, all but two physicians in the emergency department and all physicians on the wards were blinded, but since two physicians in the emergency department had to record which patients were seen by a medical student and which by a physician alone, there might have been a Rosenthal-effect (a self-fulfilling prophecy).

The length of stay was somewhat overestimated, since the entry time sometimes does not reflect the actual arrival of the patient in the emergency department but the time of the initial telephone call by the rescue coordination center. This flaw randomly affected patients in both groups, however, we cannot fully exclude that the length of stay in the emergency department was longer for the patients seen by the students.

Thirdly, the patients were not randomized. However, the medical students saw older patients and there was a non-significant trend towards the higher PCCL (patient comorbidity complexity level) for the patients seen by the supervised medical students, so one can assume that the difference in diagnostic accuracy is not attributable to an allocation of the sicker patients to the emergency department physicians.

In conclusion, given that close supervision is provided, medical students can be involved in the evaluation process of hospitalized medical patients without disadvantage for the patients in regard to diagnostic accuracy. Further studies are warranted to examine the effect of student involvement in patient care in order to improve the learning benefit for the students, the workload for the doctors and the outcome for the patients.

## Materials and Methods

### Study design

We conducted a controlled prospective trial. Patients were assigned for evaluation by an emergency department physician working alone or to a supervised medical student.

In the emergency department one physician closely supervised up to four final year medical students (Supervisor) but was not involved in patient care directly. The other physician directly cared for all the patients who were not assigned to student history taking.

**Figure 1:**
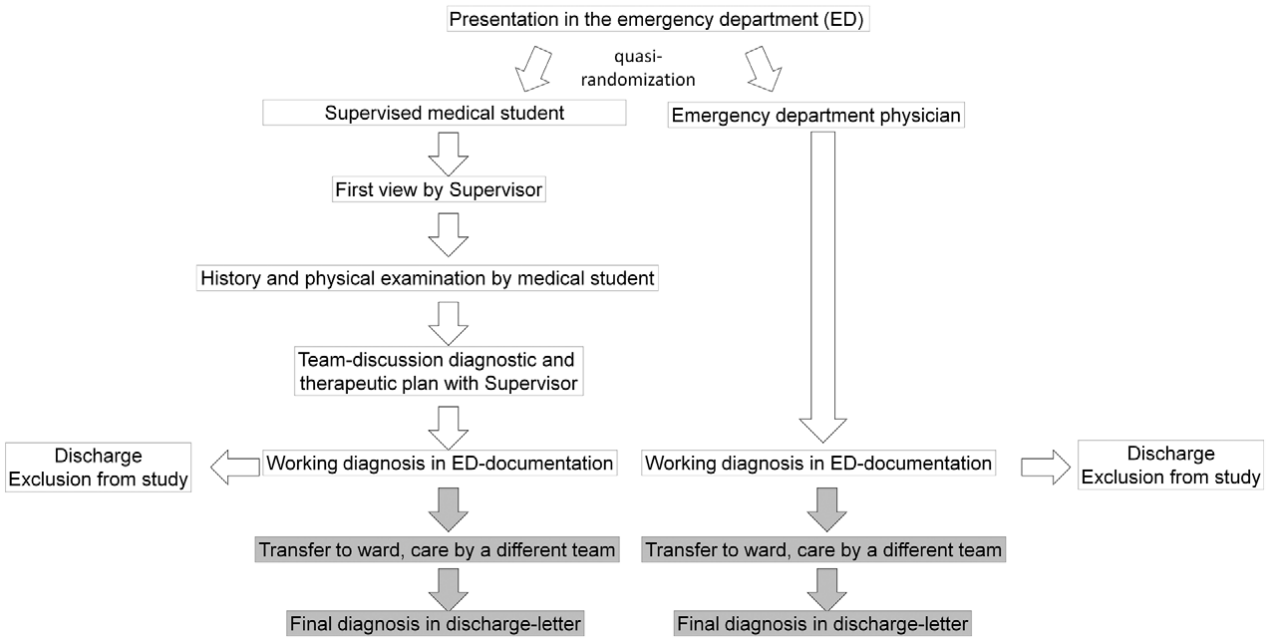
Flow-chart of the study design.

Student history taking was not completely repeated by the Supervisor, but the Supervisor was free to take selective aspects of the history as he or she saw fit. The flow-chart of the study design is shown in figure 1.

### Setting

About 8500 patients present to our medical emergency department each year. The university hospital has the only emergency department specialized in internal medicine in the health care district and also serves as a primary care facility in addition to providing the specialized care. Since ambulatory emergency patients are covered by general practitioners and surgical emergency patients are taken care of by the surgery emergency department, sick and multimorbid medical patients, who require hospitalization constitute quite a large proportion of the patients presenting in our medical emergency department. Approximately 40% of the patients presenting in the medical emergency department are admitted for further treatment to the wards and a further 28% of the patients are admitted to the clinical decision unit and discharged within 24 h.

The emergency department was staffed with two physicians and two to four final year medical students from November 2010 to March 2011.

### Selection of Participants

The medical students (n = 41, 20 m, 21 f) were in their final year without previous clerkships in the medical emergency department. They worked four weeks in the medical emergency department.

The physicians (n = 22, 12 m, 10 f) had graduated at least four years ago and had worked in the medical emergency department for at least six month.

All students, most of the physicians (n = 20, 91%) in the emergency department and all the physicians on the wards were blinded in regard to the study question. The remaining two physicians in the emergency department recorded which patients were seen by a supervised medical student and which by an emergency department physician.

From November 2010 to March 2011 all patients who presented in the medical emergency department during the day shifts on weekdays with two physicians staffing the emergency department were assigned to evaluation by either a supervised medical student or an emergency department physician. There were no selection criteria, the patients were allocated to the first physician available, either the emergency department physician working alone or the Supervisor working with the medical students. We included only non-critically ill patients (encoded “yellow”, “green” or “blue” according to the Manchester triage system, “orange” if the Supervisor thought that student involvement was possible without endangering the patient) [39], [40]. Critically ill patients were transferred to the intensive care unit and cared for by another team of physicians, so neither the emergency department physician nor the Supervisor evaluated patients who could not be seen by medical students. Only patients who were admitted (n = 323) and thus cared for by another team of physicians after the transfer from the emergency room to the ward or clinical decision unit for the same problem were included in the study.

### Intervention: Supervision

Patients who were assigned to history taking by a medical student were cared for as follows:

Concomitantly to the nurses' patient triage by the Manchester Triage system the Supervisor assessed all patients assigned to history taking by the supervised medical student. If vital danger was imminent, the Supervisor immediately admitted the patient to the intensive care unit. If not, the medical student took over, drew blood and performed history taking and physical examination. Then the medical student devised diagnostic and therapeutic plans and discussed them with the other students and the Supervisor. Then the Supervisor ordered the diagnostic procedures and prescribed the medication as discussed. In addition, the Supervisor oversaw and gave feedback on selective aspects of history taking, physical examination and manual procedures and aided the students with the documentation and administration. It was also the Supervisor's responsibility to ensure the timely manner of diagnostic and therapeutic procedures for the sicker patients. The staff contacted the Supervisor in every question concerning the patients cared for by the supervised medical student. At the end of the evaluation process, the medical student completed the documentation sheet with the working diagnosis as discussed, the Supervisor revised and signed the documentation. At that point the diagnosis was based on the data collected during history taking, the physical examinations, sometimes the referral letters, the laboratory results, the ecg and – if requested- imaging studies (echocardiography, ultrasound, computertomography).

Both medical emergency department physician alone and Supervisor were free to contact consultants as they saw fit.

### Methods of measurement and outcome measures

Our primary outcome was the diagnostic accuracy defined as the congruence between working diagnosis from the emergency department and the final diagnosis from the discharge letter from the ward or clinical decision unit. The emergency department physician and supervised medical student in the emergency department formulated a working diagnosis as a free text at the end of the emergency department admission documentation. We compared the working diagnosis with the final diagnosis in the discharge letter from the ward or clinical decision unit (also free text). The rater was blinded to the intervention. The diagnoses were deemed congruent if the wording in the discharge letter was identical or very similar to the working diagnosis in the emergency department documentation. The diagnoses were deemed not congruent, if the diagnosis was obviously different (e.g. pneumonia instead of pulmonary embolism) and required a different therapy or if the wording was similar, but with a negation (e.g. working diagnosis: acute coronary syndrome, final diagnosis: exclusion of acute coronary syndrome).

If the congruence was not clear (in 30/310 patients, 9,7% of the cases), a blinded medical student and a blinded emergency department physician discussed the case and agreed on whether the working and final diagnoses were congruent or not.

### Data collection and processing

The following information was gathered from the hospital information system:

Working diagnosis from the emergency department documentation.
Final diagnosis from the discharge letter.
Age of the patients.
Length of stay in the emergency department.
PCCL-Level (Patient comorbidity complexity level, an index which modifies the reimbursement according to the severity of the patients comorbidities in our national Diagnosis related Group (DRG)-System).
Diagnostic procedures requested in the emergency department.

From the diagnostic procedures requested in the emergency department we calculated the approximate expenditures for diagnostic procedures using the EBM-registry (“Einheitlicher Bewertungsmaßstab”), the official national medical fee schedule for hospitals in Germany. Laboratory tests and ECG were not included, since every patient received a laboratory test directed at the cardinal symptom and an ECG.

### Primary data analysis

We used JMP 9.0 (SAS Institute, Cary, North Carolina, USA) for the statistics. Non-parametric data were compared using Fisher's exact test. Parametric variables had skewed distributions. Therefore, differences were tested using the Wilcoxon-Test. For all tests, a two-tailed alpha of 0.05 was used.

### Ethics

The study-protocol was approved by the ethics committee of the University of Tuebingen, decision number 488/2010A.
